# Four Remarkable Cases: Genito-Urinary Sporotrichosis in New Zealand

**Published:** 1915-04-10

**Authors:** 


### FOUR REMARKABLE CASES.

### Genito-Urinary Sporotrichosis in New Zealand.

There is a danger at the present time of clinical records of interest, apart from those of the Royal Army Medical Corps, being overlooked. The following, however, must not be crowded out of memory. In the *Australasian Medical Gazette* (June 6, 1914, p. 497) Professor S. T. Champtaloup, of Otago University, described four remarkable cases of genito-urinary sporotrichosis from the Auckland district, in each of which the clinical condition was very strongly suggestive of tuberculosis. In all the cases the organism isolated was the *Sporotrichum Goujeroti*, first described by Goujerot in 1907; the first definite case of sporotrichosis in Great Britain was published by Walker and Ritchie in 1911. Hitherto but one case of renal sporotrichosis has been described, by Rochard, Duval, and Bodolec in 1909.

The first of Professor Champtaloup's four cases was that of a man who came under medical observation in 1905, but had been troubled with bladder symptoms for some years before that. At this time he had symptoms of cystitis, thought to be tuberculous. Cystoscopic examination showed tuberculous-looking ulceration round the orifice of the right ureter and for some distance along the trigone. The right kidney was probably enlarged, and the left ureter appeared normal. Three specimens of urine yielded no tubercle bacilli, nor did an inoculated guinea-pig develop tubercle. Cultures gave a growth of sporotrichum. The patient was treated with iodides, which he tolerated badly: he left the district and had other treatment elsewhere, but still suffers from the disease. The second case was that of a man who in 1913 had attacks of pain referred to the right kidney, with frequency of micturition and pyuria. A guinea-pig inoculation was negative. A surgeon removed the appendix, which, unfortunately, was not examined; but symptoms persisted, and later the sporotrichum was isolated from the urine. Under large doses of iodides rapid improvement followed, and the patient soon returned to work. The third patient, a woman, also had her appendix removed for right-sided pain, and the uterus was suspended. A year later the right ovary was removed, again without relief. Later still an exploratory laparotomy was performed, and many adhesions were separated. Pain returned, and tuberculosis of the kidney was suspected; but the case turned out to be one of sporotrichosis. Potassium iodide caused great improvement, but the patient has since been lost sight of. The fourth patient, another female, had attacks of backache and hæmaturia off and on for nine years. Examination with *x*-rays and with the cystoscope and inoculation of guinea-pigs were negative; but a specimen of urine taken from the right ureter was found to contain the *sporotrichum*. Marked benefit has ensued from hydriodic acid (the patient proved intolerant of potassium iodide). These cases show the danger of making a positive diagnosis of vesical tuberculosis from cystoscopic appearances only. It will be of interest to see whether, now that attention has been directed to the matter, cases of urinary sporotrichosis will be reported in Europe.

